# Supplementary material for: Venomix: a simple bioinformatic pipeline for identifying and characterizing toxin gene candidates from transcriptomic data
Source: PeerJ. 2018 Jul 31;6:e5361. doi: 10.7717/peerj.5361 (PMC6074769; doi:10.7717/peerj.5361)
Supplement: Supplemental Information 6 [file peerj-06-5361-s006.gz › FinalOutput_1E-6/Snake_venom_metalloproteinase_Ac1_1/finaltree.pdf]

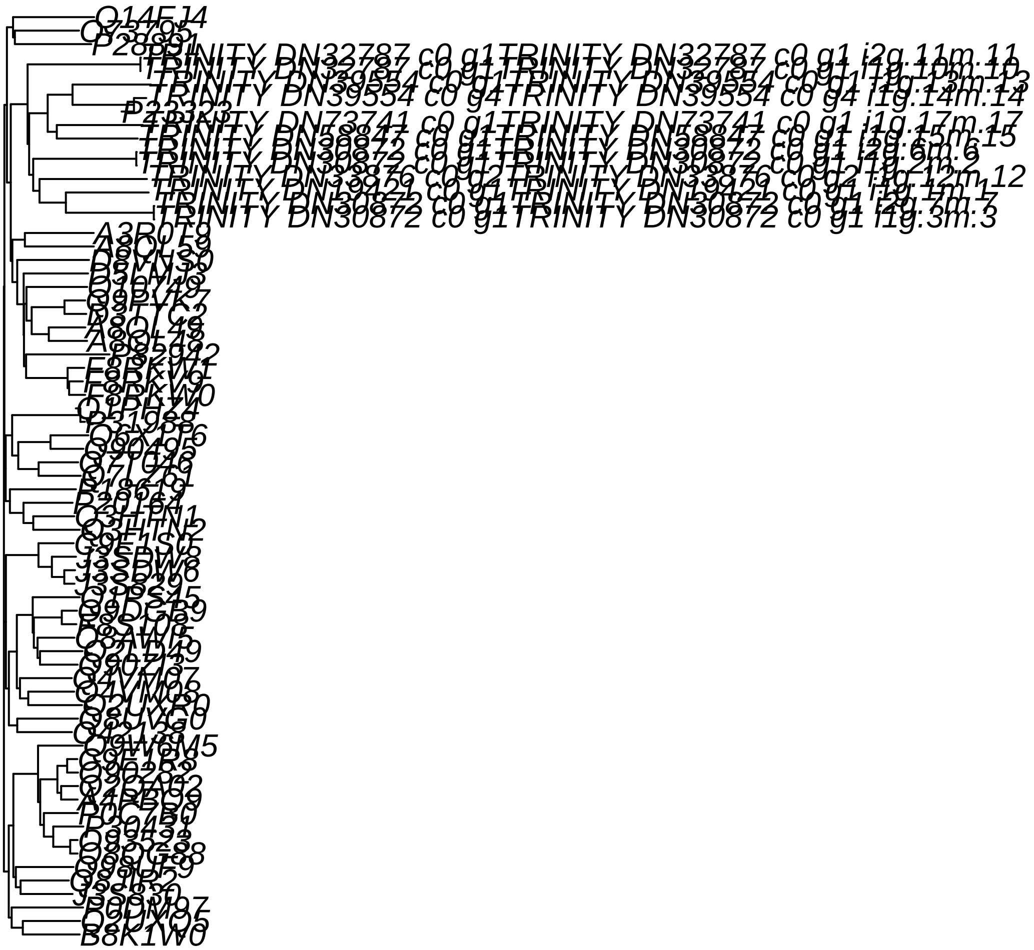
Q14FJ4
TRINITY DN32787 c0 g1TRINITY DN32787 c0 g1 i2g.11m.11
TRINITY DN39554 c0 g4TRINITY DN39554 c0 g4 i1g.14m.14
TRINITY DN73741 c0 g1TRINITY DN73741 c0 g1 i1g.17m.17
TRINITY DN30872 c0 g1TRINITY DN30872 c0 g1 i1g.3m.3
A3R0T9
B8K1W0
